# The emotional effects on professional interpreters of interpreting palliative care conversations for adult patients: A rapid review

**DOI:** 10.1177/02692163231169318

**Published:** 2023-04-24

**Authors:** Jennifer A Hancox, Clare F McKiernan, Alice L Martin, Jon Tomas, John I MacArtney

**Affiliations:** 1 Department of Supportive and Palliative Care, University Hospitals Birmingham, Birmingham, UK; 2 Department of Supportive and Palliative Care, Birmingham St Mary’s Hospice, Birmingham, UK; 3 Department of Supportive and Palliative Care, University Hospitals of Coventry and Warwickshire, Coventry, UK; 4 Unit of Academic Primary Care, University of Warwick, Coventry, UK

**Keywords:** Health personnel, palliative care, terminal care, psychological distress, systematic review, professional interpreter

## Abstract

**Background:**

Professional interpreters working in palliative contexts improve patient care. Whilst literature identifies psychological distress in other healthcare professionals, research into emotional effects on professional interpreters in this highly emotive setting is limited. Isolating emotional responses may enable targeted interventions to enhance interpreter use and improve wellbeing. Timely evidence is needed to urgently familiarise the profession with issues faced by these valuable colleagues, to affect practice.

**Aim:**

Describe the emotional effects on professional interpreters of interpreting adult palliative care conversations. Collate recommendations to mitigate negative emotional effects.

**Design:**

We performed a rapid review of studies identifying emotional effects on professional interpreters of interpreting adult palliative conversations. Rapid review chosen to present timely evidence to relevant stakeholders in a resource-efficient way. Thematic analysis managed using NVivo. Quality appraisal evaluated predominantly using CASP checklists. Reported using PRISMA guidelines. PROSPERO registration CRD42022301753.

**Data sources:**

Articles available in English on PubMed [1966–2021], MEDLINE [1946–2021], EMBASE [1974–2021], CINAHL [1981–2021] and PsycINFO [1806–2021] in December 2021.

**Results:**

Eleven articles from the USA (5), Australia (3), Canada (2) and UK (1). Eight interview-based, two online surveys and one quality improvement project. Themes included (1) Identifying diversity of emotional effects: emotions including stress, discomfort, loneliness. (2) Identifying factors affecting interpreters’ emotional responses: impact of morals, culture and role expectations; working with patients and families; interpreter experience and age. (3) Recommendations to mitigate negative emotional effects: pre-briefing, debriefing and interpreter/provider training.

**Conclusion:**

Professional interpreters experience myriad emotional responses to palliative conversations. Role clarity, collaborative working and formal training may alleviate negative effects.

**What is already known about this topic?**

- Professional interpreters improve health outcomes in patients with Limited English Proficiency.
- Professional interpreters are often expected to interpret distressing conversations in palliative care settings yet research into subsequent emotional effects is limited.

**What this paper adds?**

- Professional interpreters experience distress, overwhelm, guilt, loneliness and discomfort when interpreting palliative care conversations.
- This review highlights that moral conflict and distress result when asked to balance professional obligations of interpreting accurately against delivering culturally sensitive information, particularly when role expectations are unclear.
- Improving clinician and interpreter relationships and communication skills may be key to targeting negative effects.

**Implications for practice, theory, or policy**

- We recommend formal guidelines clarifying the interpreter’s role and reinforce pre-briefing, debriefing and training focusing on collaborative working.
- Further research needed assessing influence of language and cultural dynamics on emotional effects.

## Background

The world is seeing an ongoing rise in international migration.^1^ Over 7000 languages are spoken worldwide,^2^ with increasing numbers of patients with language barriers within health systems of their destination countries.^1^ Language barriers are associated with reduced comprehension of medical conditions, increased drug reactions,^3^ reduced access to healthcare^4,5^ and screening services.^6^

Irrespective of language used, good communication at the end-of-life improves patient satisfaction and reduces aggressive medical interventions and hospital admissions.^7^ Patients must be given information in an understandable way, with arrangements to meet language and communication needs.^8^

A cross-cultural approach including interpreter use in end-of-life care improves ethnic minority experience.^9^ Using professional interpreters in palliative care leads to reduced healthcare access inequality, better clinical outcomes, greater patient satisfaction and potentially improved symptom management.^10^ Ad hoc interpreters are associated with higher communication error rates,^11,12^ yet literature reports inadequate access to and utilisation of professional interpreters in clinical practice.^11,13^

Research demonstrates interpreters in many sectors (including law, mental health, refugees, public services) exposed to clients’ traumatic material can experience vicarious trauma and other negative psychological consequences.^14,15^ Studies advocate a need to address a disappointing lack of support and appreciation from service providers for the complex, nuanced role interpreters provide.^15,16^ Interpreting organisations recognise this potential for vicarious trauma and burnout when exposed to challenging, emotional scenarios,^17,18^ yet support is lacking in many contexts.^15,16^ Ability to manage negative emotions may protect from compassion fatigue or adverse psychological consequences.^19^

Palliative care settings can be highly emotive for healthcare professionals, with risk of burnout and psychological distress.^20,21^ Professional interpreters may be asked to interpret in these potentially traumatic situations, including discussions around dying. Much existing research into professional interpreters within palliative care focuses on patient outcomes,^10,11,22^ yet research into the emotional effects on this cohort is limited. Emotional effects include the feelings that these conversations generate, which could significantly impact interpreters’ personal and professional lives. To our knowledge, there has been no attempt to consolidate this evidence base. Evaluating emotional effects could guide targeted interventions improving interpreter wellbeing, retention and effectiveness.

The Institute of Translation and Interpreting recently released a position statement on vicarious trauma in interpreters, recognising exposure to challenging, emotional scenarios. Organisations should be proactive in ensuring interpreters working in ‘potentially traumatising settings’ are informed of risks, briefed, debriefed and offered available support services.^17^ Given the benefits of professional interpreters and potential consequences of burnout and psychological distress, this review seeks to provide a synthesis of the emotional effects on interpreters of conducting palliative care conversations, and make recommendations to enhance interpreter experience. A rapid review has been utilised to highlight findings to stakeholders in a timely, resource-efficient way, to influence institutional practice and policy on working with interpreters in palliative care.

## Aims

The aim of this rapid review is to address the following questions:

1. What emotional effects upon professional interpreters have been identified from interpreting palliative care conversations for adult patients?
2. What recommendations have been made to mitigate any potential negative emotional effects in this context?

The research questions were developed using the Population, Intervention, Comparison, Outcomes and Context (PICOC) framework (Table 1).

**Table 1.** PICOC criteria used to develop research questions.

Population	Professional interpreters
Intervention	Palliative care conversations in adult patients
Comparison	Emotional effects
Outcomes	Emotional effects of palliative care conversations on professional interpreters
	Recommendations to mitigate negative emotional effects
Context	Any healthcare setting

## Methods

### Design

This review employs rapid review methodology^23^; an approach to synthesising knowledge in a shorter timeframe by simplifying or excluding aspects mandated in traditional systematic review. A rapid review was chosen for its ability to present timely evidence to relevant stakeholders (palliative care professionals, educators and interpreting agencies) in a resource-efficient way,^24^ to affect change in current institutional practice. This was in the context of time and resource limitations of our research team, predominantly full-time clinicians when this review was undertaken. Elements simplified included limiting search terms, limiting publication language to English, excluding grey literature and supplemental searches.^24^ The study selection, quality appraisal and data synthesis were predominantly performed by one researcher, with other authors reviewing and discussing at each stage. The decision to simplify these aspects was made by the research team to make the review manageable, whilst maintaining sufficient quality.

The review is reported using PRISMA (Preferred Reporting Items for Systematic Review and Meta-Analyses) guidelines.^25^ The project was registered on PROSPERO in January 2022, with amendments made February 2022 and is available at https://www.crd.york.ac.uk/prospero/display_record.php?RecordID=301753.

### Search strategy

A search of PubMed [1966–2021], MEDLINE [1946–2021], EMBASE [1974–2021], CINAHL [1981–2021] and PsycINFO [1806–2021], was conducted in late December 2021. Searches were designed for each database using free text and MeSH terms. Free text terms relating to ‘palliative care’ (palliative medicine, end-of-life, terminal, death, dying, supportive care, terminally ill) AND ‘interpreter OR translator’ were utilised. MeSH terms for each database were applied for example, ‘palliative care’, ‘terminal care’, ‘death and dying’ (Supplemental File 1). Search terms were agreed following group consensus amongst researchers, alongside discussion with an experienced information specialist at the clinical library, to identify broad themes fitting our review aims.

### Eligibility

Eligibility for analysis was based on inclusion and exclusion criteria (Table 2).

**Table 2.** Inclusion and exclusion criteria.

*Inclusion criteria*: 1. Palliative care conversations, including but not limited to, goals of care, breaking bad news, prognosis, diagnosis, death and dying 2. Study including professional interpreters (as described by paper) interpreting palliative care conversations for adult patients and/or their families 3. Studies that document the emotional effects of interpreting palliative care conversations on professional interpreters
*Exclusion criteria*: 1. Studies looking solely at the use of non-professional interpreters (family members, staff not trained in professional healthcare interpreting) 2. Studies that do not comment on the emotional effects on the interpreter of palliative care conversations 3. Commentaries, opinion pieces, non-peer reviewed articles or grey literature 4. Articles not available in English

### Study selection

One researcher (JH) screened article titles and abstracts to assess for inclusion. A second researcher (AM) reviewed the study selection process and supported decision making. Articles meeting criteria were retrieved for full text review. Articles where researchers (JH and AM) agreed it was unclear from the title/abstract whether the study met criteria were also retrieved. Where there was uncertainty, articles were discussed with the remainder of the research team. Mendeley software was used for organising articles.

### Data extraction

A Microsoft Excel proforma was used for data extraction, capturing: author, year, journal, funding, country, aim, design, setting, interpreter characteristics, key findings and conclusions/author recommendations relating to our research questions. Two researchers (JH and CM) independently extracted data before comparing for agreement.

### Quality appraisal

Quality appraisal was performed to assess validity and relevance of studies for inclusion, ensuring findings were of sufficient usefulness to our research questions. Qualitative studies were appraised by one researcher (JH) according to CASP checklists,^26^ tools designed to critically appraise study designs. Outcomes discussed with a second researcher (JM) for agreement when unclear. Cross-sectional studies were evaluated using the AXIS appraisal tool,^27^ and the quality improvement project appraised using the QI MCQS.^28^

### Data synthesis

Articles were imported into Nvivo and one researcher (JH) coded data using Braun and Clarke’s^29^ principles for thematic analysis. Findings relevant to our research questions in ‘Results’ sections of articles reviewed were coded, including author findings, participant quotes and tables/diagrams (the latter where relevant findings were not referenced in the main text). Quantitative data were coded where any statistical finding met our aims – as described by authors in ‘Results’ sections or presented in tables. This process involved familiarisation with extracted data, expanded further with rereading of the texts, allowing initial themes to be identified deductively through exploration of aims and study findings. Examples of codes used within broader themes included descriptions of emotional effects such as discomfort, distress and guilt.

Our analysis recognises the cultural boundedness of emotions and the analysis of the original researchers, and that we should not assume the terminology used is consistent across cultures. We therefore collated the range of emotions reported in the papers and provide linguistical and national contexts that may suggest potential differences. Situational factors which might influence the emotional response, such as interpreter experience, were also identified. Once data were assigned to codes, themes were reviewed before categorisation and subdivision. Thematic reports were discussed amongst all researchers to check agreement and refine interpretation. A thematic analysis was used as its theoretical freedom allowed us to generate a rich, detailed account of issues faced by professional interpreters, with sufficient complexity to interpret findings prompting policy change and further avenues for research. A thematic analysis approach is an efficient way to conduct a rapid review, as each stage (familiarisation; generating codes; constructing themes; revising; defining themes; writing-up)^29^ are necessary steps in a qualitative rapid review process.

### Results

**Figure 1.**
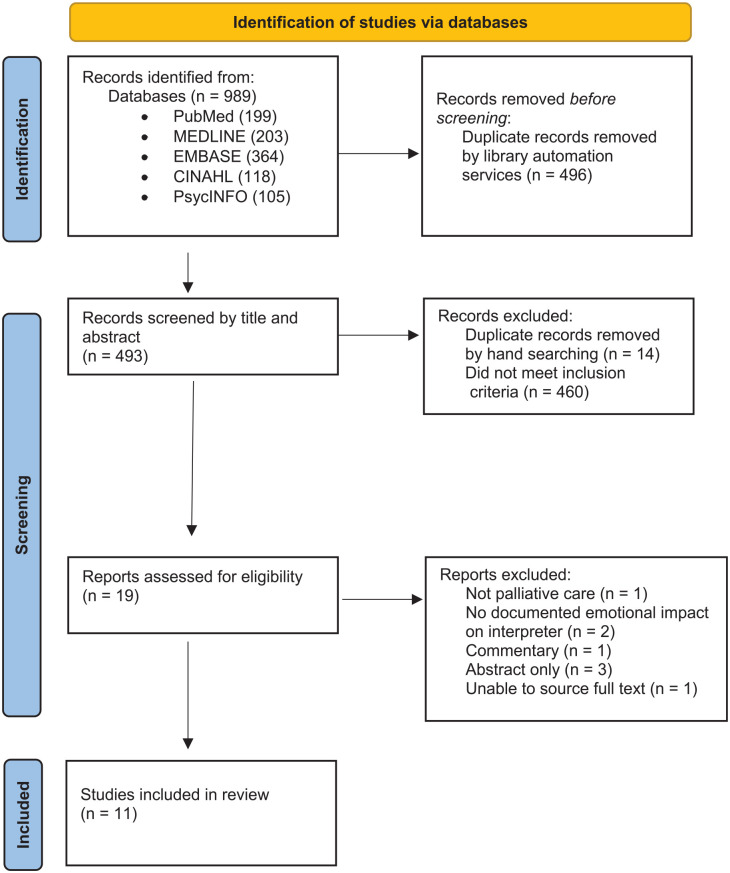
PRISMA checklist.

Nineteen articles were identified for full text retrieval, of which 11 were included for analysis (Figure 1).

### Study characteristics

Table 3 summarises key characteristics from included studies.

**Table 3.** Study characteristics.

Years published	1999–2021	
Country of study	USA^30–34^	5
	Australia^35–37^	3
	Canada^38,39^	2
	UK^40^	1
Languages	Spanish Only^30^	1
	Spanish and Chinese^32^	1
	Inuit (Inuktitut, French)^38^	1
	Aboriginal (Cree/Ojibway)^39^	1
	More than 2 languages^33,34,37,40^	4
	Unspecified^31,35^	2
Study design	Qualitative interviews/Focus groups with interpreters^30,32,34,37,38,40^	6
	Interviews with healthcare professionals^36^	1
	Observational/Interview data from patients, relatives, healthcare professionals and interpreters^39^	1
	Cross-sectional surveys^33,35^	2
	Quality improvement project^31^	1

Whilst most were qualitative studies,^30,32,34,37,38,40^ two articles were cross-sectional online surveys distributed to healthcare interpreters.^33,35^ One article described a quality improvement project evaluating an intervention to improve interpreter confidence in palliative care conversations.^31^ One article was part of a series of work on Aboriginal Canadians – included due to extensive use of emotive language.^39^

When participant gender was reported, there was a higher proportion of female interpreters.^30,32–35,37^

### Quality appraisal results

All articles meeting inclusion/exclusion criteria were appraised, using tools appropriate for their study design, to guide inclusion in our review. All articles were of sufficient quality to be useful in answering our research questions and were therefore included for analysis (Supplemental File 2).

### Results of individual studies

Key features of individual studies are detailed in Table 4.

**Table 4.** Results of included studies.

Authors, year, country	Aim	Study design, setting	Key interpreter characteristics	Findings and author recommendations *(in relation to research question)*
Rhodes et al., 2021, USA	1. Better understand the challenges professional medical interpreters face and how they affect the accuracy of provider-patient communication during discussions of end-of-life	Qualitative, semi-structured interviews 2 academic medical centres	*n* = 17 *Language*: Spanish *Female*: 82.3% *Age*: 42.7 (mean) *Experience (years)*: 11.1 (mean) *National certification*: 94.1% *EOL discussion frequency*: < monthly (47.1%), weekly to monthly (52.9%)	• Spanish interpreters focus on accurate translation even if led to personal distress. • Rare alterations in flow and content may result from lack of conversational space, provider language, provider lack of empathy. • Interpreters impacted by emotional and professional distress. Authors recommend provider and interpreter training/support. Further research to explore effect of emotional modulation by interpreters on patients and whether findings translate to other contexts.
Goldhirsch et al., 2021, USA	1. Assess the effectiveness of a six-session monthly dialogue-based course on improving interpreter confidence in palliative care conversations	Quality Improvement Project 2 hospitals	*n* = 44 completing pre and post questionnaires (50 completed course)	• Interpreters showed significant increase in post-intervention confidence in palliative care communication and valued physician collaboration and ‘huddles’. • Interpreters described stress relating to challenges interpreting end-of-life vocabulary and violating cultural norms. Authors comment on ongoing delivery of intervention.
Silva et al., 2020, USA	1. To develop insights from medical interpreters about their role when interpreting discussions regarding goals of care and end-of-life issues 2. Identify practices interpreters perceive as helping to improve or hinder patient-provider communication 3. Obtain suggestions on how to improve communication during these conversations with Spanish and Chinese-speaking patients	Qualitative, semi-structured interviews 1 large tertiary hospital	*n* = 12 *Language*: Spanish (7), Chinese (5) *Number of non-English languages spoken*: 1 (50%), 2 (25%), 3+ (25%) *Female*: 58% *Age*: 45.6 (mean) *Certification*: 100% *Training* >*40h*: 75% *Experience (years)*: 5.4 (mean) *EOL discussion frequency*: 2.7 h/week (mean)	• Participants felt comfortable delivering end-of-life messages although subset needed to compartmentalise emotions. • Expectation to provide literal interpretation but also act as cultural broker, which can result in internal conflict. Authors recommend conducting pre-meetings, debriefing and provider/interpreter training. Also recommend greater clarification of interpreter role.
Schenker et al., 2012, USA	1. Assess the experiences of healthcare interpreters when interpreting discussions about end-of-life issues 2. Identify interpreter characteristics and experiences that may be associated with improved satisfaction and comfort with interpreting these discussions 3. Describe interpreter training needs	Descriptive, cross-sectional, national online survey Mixed settings	*n* = 142 *Language*: Multiple, including Spanish (73%) *Female*: 81% *Age*: 46.2 (mean) *Certification*: 71% *Training* >*40h*: 89% *Experience (years)*: >5 (65%) *EOL discussion frequency*: None 14.7%, Some 85.3% (most multiple discussions/week) Multiple interpretation modalities	• Most interpreters have experience with end-of-life discussions but only half report these go well. • When interpreters feel clear about their role, they are more likely to feel comfortable. • Interpreters experience emotional stress from interpreting and would value more training for themselves/providers. Authors recommend training and emotional support for interpreters. Further research to examine effect of training elements and different interpreter roles on interpreter, patient and clinician outcomes.
Norris et al., 2005, USA	1. Improve understanding of how to approach discussions between language-discordant patients and clinicians about terminal or life-threatening illness	Qualitative, focus groups Setting not specified (likely mixed)	*n* = 43 (Group 1) *Languages*: Multiple (26) *Number of non-English languages spoken*: 1 (64%), 2 (21%), 3 (12%) *Country of origin*: Asia (39.5%), N America (30.2%), S/C America (16.3%), Europe (11.6%), Africa (2.3%) *Female*: 65% *Age*: 49 (median) *Training* >*40h*: 100% *Experience (years)*: 6 (median) *n* = 25 (Group 2) *Country of origin*: Asia (60%), N America (20%), S/C America (16%), Africa (4%) *Female*: 64% *Age*: 51 (median) *Experience (years)*: 10 (median)	• Interpreters experience struggle of role conflict (strict interpretation versus cultural broker). • Interpreters recommend pre-meetings and role clarification. Authors recommend supporting the use of interpreters as cultural brokers providing clinicians are aware and supportive. Further research into challenges faced by interpreters in end-of-life care and recommendations made by interpreters.
James et al., 2021, Australia	1. Assess the impacts of difficult conversations, defined as discussions involving explanation of a serious diagnosis, goals of care or death/dying in increasing risk of compassion fatigue in healthcare interpreters 2. Discover interpreters’ opinions on ways to improve these conversations	Descriptive, cross-sectional, regional online survey Mixed settings	*n* = 94 *Female*: 71.3% *Age*: >40 (76.5%), 18–30 (8.5%) *Experience*: >10 years (59.6%) *National Accreditation*: 84% *EOL discussion frequency*: < once/month (50%)	• Interpreters were not experiencing compassion fatigue. • Younger age and less experience associated with higher stress/burnout scores. • Various contributors to conversation difficulty described and recommendations to mitigate this. Authors recommend provider/interpreter training including pre-briefing, and formal debriefing. Further research to identify characteristics predisposing to compassion fatigue.
Martin et al., 2020, Australia	1. Explore the views of healthcare professionals regarding working with interpreters in a cancer setting	Exploratory, cross-sectional qualitative, focus groups and semi-structured interviews 1 tertiary hospital (covering hospital and community services)	N/A [25 HCPs – 14 physicians, 4 nurses, 7 allied health]	• Healthcare professionals have some awareness of issues faced by non-English speaking patients and interpreters including emotional impact and cultural issues. Authors recommend further training/support for healthcare professionals and interpreters in end-of-life discussions and a greater appreciation for their respective challenges. Also recommend routine pre-briefing and debriefing.
Kirby et al., 2017, Australia	1. Explore the experiences and perspectives of professional interpreters in supporting the transition of culturally and linguistically diverse patients to specialist palliative care	Qualitative, semi-structured interviews 2 metropolitan hospitals	*n* = 20 *Languages*: Multiple *Number of non-English languages spoken*: 2 (1), 3 (1) *National Accreditation*: 90% *Female*: 85%	• Interpreters face a range of interpersonal and interprofessional challenges. • Recognition of these may help provide support and improve healthcare professional-interpreter interactions. Authors recommend further training/support for interpreters and doctors, including doctor awareness of emotional demands on interpreters and utilisation of interpreter expertise.
Hordyk et al., 2017, Canada	1. Understand the experience of Inuit interpreters in the context of end-of-life care in Nunavik in order to identify training needs	Qualitative, formal and informal interviews. Part of larger study using focused ethnographic methodology Local health centres, Montreal tertiary care contexts	*n* = 24 *Languages*: Inuktitut, French, English [103 participants in larger project included nurses, physicians, social workers, interpreters, spiritual advisors]	• Inuit interpreters work with little training in the context of multiple linguistic and cultural challenges and emotional stress. • Interpreter training is needed to support linguistic/moral/ethical dilemmas in circumpolar contexts. • Provider training need to improve cultural awareness. Authors recommend interpreter/provider training. Further research to identify if educational interventions improve interpreter retention and communication satisfaction in end-of-life care.
Kaufert, 1999, Canada	1. Examine the process of cultural mediation in end-of-life care of Aboriginal people in urban hospitals	Qualitative, interviews and observational data General medical/palliative care wards of 2 large teaching hospitals	*n* = 8 *Languages*: Cree/Ojibway (Aboriginal Canadian)	• Multiple linguistic/cultural dilemmas faced by interpreters. Interpreter as key to mediate conflicting cultural and ethical values in end-of-life discussions.
Prentice et al., 2014, UK	1. Identify challenges faced by professional translators 2. Identify whether a larger, multicentre study is warranted and feasible	Exploratory, qualitative, semi-structured interviews Regional cancer centre (outpatient)	*n* = 5 *Languages*: BSL (2), Bengali/English (2), Polish/English (1) *Age*: 23–60 *Experience (years)*: 2–17	• Significant emotional impact on interpreters of interpreting end-of-life discussions. • Absence of formal training/support for interpreters. Authors recommend increased access to interpreter training, support, debrief and healthcare professional education to understand interpreter role. Further research to dissect translation from advocacy, breadth of interpreter roles and establish training/support needs. Research challenges facing translators on large scale.

## Results of collation and syntheses

### Identifying diversity of emotional effects of interpreting palliative care conversations

A spectrum of emotional responses in professional interpreters was identified, encapsulating the stress of the role, deep emotional distress, guilt of conveying bad news and loneliness. Experiences of job satisfaction and community service were also recorded.

Studies described stress,^30,31,33,36,38^ sadness/upset,^40^ distress^38,40^ and feeling overwhelmed.^32,38^ An American survey reported 76% of interpreters found end-of-life conversations more stressful than others, and a substantial proportion found discussions emotionally overwhelming.^33^ However, an Australian survey reported much lower rates of stress, with all respondents identifying low (84.8%) or moderate (15.2%) burnout, and low (83.5%) or moderate (16.5%) Secondary Traumatic Stress scores; a subset of younger and less experienced interpreters reported higher scores.^35^ Most interpreters in both studies had over 5 years’ experience and a form of training certification.^33,35^ Most interpreters in the former study interpreted end-of-life discussions multiple times a week^33^; in the latter, 50% interpreted difficult conversations less than monthly.^35^

Interpreting palliative care conversations involved ‘emotional burden’,^36,37^ ‘emotional toll’,^34^ ‘trauma’^37^ and ‘deep emotional distress’.^38^ Healthcare professionals in Australia suggested this burden may be related to a patient’s decision-making, or transference.^36^ Other studies referred to these feelings in the context of professional interpreters resolving their professional identity^37^ and regulating conflict.^38^ One study (*Multiple languages, UK*) identified an emotional connection from being the first to both relay news to patients and receive the response.^40^ Interpreters described significant distress when patients died, an experience like the death of a loved one.^40^ Behaviours indicating empathy or sympathy were often described.^30,32,37,38,40^

*I would really take that home with me I would be really really upset and um really worried for the person. (Interpreter, UK)* ^40^

*. . .It’s primal fear. Some people, it’s their death, but some people is knowing their kids will be without a mum or without a dad and I can relate to that. (Interpreter, Australia)* ^37^

Interpreter discomfort was identified, referring to feelings of unease and awkwardness leading to dissatisfaction with encounters.^30,33,34,36–38^ This was associated with relaying messages conflicting with cultural, social or personal expectations of communication, or when interpreters felt they wanted to expand or clarify the underlying message.^30,37,38^ Interpreter discomfort was also inferred in an article exploring cultural mediation in Aboriginal Canadians.^39^ Interpreters felt ‘forced. . .into developing reductionist, decontextualised accounts’ when providers expected immediate generalised responses to individualised cultural nuances in the context of death and dying.^39^ American survey data, however, reported only 15% felt uncomfortable interpreting end-of-life discussions, despite finding them more stressful.^33^

*Sometimes you soften things. I had a case where we walked in, doctors said, “This is your scan. You’ve got a huge tumor on your kidney. There’s nothing I can do about it” I could not do that. (Interpreter, Australia)* ^37^

‘Guilt’ was associated with the responsibility of delivering bad news or being on the receiving end of distress.^35,40^ Less frequent emotions experienced included anger at patients’ circumstances,^37^ shock at argumentative clients^38^ and horror when clinicians are disengaged from distressing conversations.^35^

*. . .she [the patient] was angry, that’s why she took out on me but still made me feel bad and upset, you know? (Interpreter, UK)* ^40^

‘Loneliness’^32,35,39^ was described, including feelings of isolation,^38^ abandonment^34^ or alienation,^31^ often when feeling disparate from clinicians or services.

*We are a forgotten breed. We’re always needed but do not have the resources to do our job. (Inuit interpreter, Canada)* ^38^

There were also reports, albeit less referenced, of positive emotions.^32,33,35^ Compassion satisfaction scores were moderate (34.1%) or high (65.9%) in Australian interpreter survey respondents.^35^ 22% of US interpreters surveyed felt end-of-life discussions were more satisfying than other conversations and 85% felt comfortable interpreting them.^33^ All 12 interpreters in one study (*Spanish and Chinese, USA*) felt comfortable delivering palliative care conversations, although they conducted them less than weekly.^32^ Interpreters supporting transition from oncology to palliative care (*Multiple languages, Australia*) described rewarding aspects, including a sense of responsibility, community service and capacity to benefit patients, although emotional benefits were not elaborated on.^37^

Behaviours potentially associated with emotional effects were detailed and help illustrate the impact these emotions have. We therefore included these findings within this theme. Interpreters reported crying,^37,38^ refusal to work with palliative care patients due to emotional burden^37^ or difficulty translating.^38^ A subset of interpreters (*Spanish and Chinese, USA*) reported needing to compartmentalise their emotions to conduct their role^32^ and quotes from another study (*Multiple languages, Australia*) described maintaining professional ‘fronts’, with displaying emotion considered unacceptable.^37^

### Interpreters in context: Identifying factors that affect interpreters’ emotional responses

The impact of morals, culture and role expectations on interpreters’ emotional responses. Most studies highlighted how interpreter translation encompasses not only language, but cultural norms and moral assumptions – each often intersecting and overlapping with the other. Their role was associated with references to strain,^30^ conflict,^32–34,39^ tension,^34^ clash,^39^ feeling caught^30,34,38,39^ and the need for negotiation.^34,37^

The term ‘moral conflicts’, from Hordyk et al. (*Inuit interpreters, Canada*),^38^ applied in some capacity in all studies. These ‘moral conflicts’ were associated with negative emotional responses. Such conflicts include the complexities, challenges and emotional consequences of differing expectations of the interpreter’s role by healthcare professionals, interpreters, patients and institutions. It encapsulates conflicting perceptions of the interpreter as a neutral conduit (interpreting verbatim what the provider dictates),^37^ cultural broker or mediator (incorporating a cultural framework for comprehending the interpreted message) or patient advocate (action on the patient’s behalf outside of interpreted interview).^39^ Deviation from the role as neutral conduit was considered when cultural or emotional sensitivity was felt lacking in the encounter.^30,32,34,37–39^ Kaufert^39^ describes this ethical dilemma in Aboriginal Canadians, whereby interpreters work within institutions dominated by bioethical principles, yet are influenced by their cultural roots and values – creating a ‘double identity’.

Moral conflicts resulted in internal dispute and emotional distress related to perceived professional obligations of accuracy balanced against the desire to deliver sensitive and culturally appropriate information.^30,32,34,37–39^ Many studies indicated some interpreters felt obliged to honour accurate translation^30,32,37–40^; in Spanish and Chinese interpreters this was even at the expense of personal discomfort.^30,32^ However, this was described by Spanish interpreters (*USA)* as ‘challenging’ or ‘unrealistic’, at times resulting in rare intentional alterations when patient emotional wellbeing was at stake.^30^ Strategies such as ‘contextualising’^32^ or clarification with healthcare professionals^37^ were utilised to adapt to cultural expectations. One study (*Multiple languages; USA*) described interpreters feeling a need to ‘protect’ the patient from the physician and stated being used only as a conduit, rather than a healthcare team member, was a ‘disservice’.^34^ Some interpreters preferred acting as cultural informants and/or advocates.^32,38–40^ Spanish and Chinese interpreters (*USA*) saw *both* literal interpretation and acting as a cultural broker as essential considerations.^32^

*The interpreter is supposed to stay away and I try to keep it that way . . . But when it comes to palliative care . . . I’ll ﬁnd that I’m the eye contact person between the patient and family and the doctor . . . ‘Cuz it’s a very sensitive topic. (Spanish interpreter, USA)* ^30^

*I have this cultural empathy . . . when there are family members with patients they just get in another state of mind, I can read that because I know the culture . . . the nuances (Spanish-speaking interpreter, USA)* ^32^

*It is so hard to tell them, how could you tell someone that you’re going to die? You can’t just tell it like that [very directly]. With our culture you don’t tell it like that. (Interpreter, Australia)* ^37^

*Hospice would roughly translate to the type of comforting care before you die . . . when interpreting those two terms [hospice and palliative care] and the description by the providers I have to do ‘contextualizing’ . . . if I don’t contextualize there is discrepancy in understanding (Chinese-speaking interpreter, USA)* ^32^

In several papers, tension resulted from violating cultural norms by maintaining strict accuracy, or potentially compromising accuracy and professional codes of ethics to act as mediator/advocate.^30–32,34,37–39^ Some studies identified the linguistic and cultural challenges of strictly interpreting ‘palliative care’ and related vocabularies.^31,32,36,37,39^ A study of Inuit interpreters emphasised this moral conflict, particularly when values of communities and healthcare professionals clashed, with no ‘right’ solution and feelings of isolation.^38^ Hospital interpreters were more likely to favour exact translation than interpreters working in smaller community contexts.^38^ Australian healthcare professionals showed insight into interpreter challenges, needing to balance accuracy and cultural sensitivity. Opinions varied regarding whether interpreters should translate verbatim or whether modification and ‘artistic licence’ were acceptable assuming the provider was informed.^36^

Clarity of their cultural and moral role helped interpreters manage the emotional effects of their work. Interpreters from America, who felt clear about their role, were more likely to think discussions went well (51% vs 11%) and feel comfortable interpreting (88% vs 56%). Interpreters feeling the doctor understood their role were more likely to think discussions went well (52% vs 22%); these experiences were not associated with satisfaction or stress.^33^ Similarly, in Australia, participants who perceived discussions favourably generally felt positively about the cultural appropriateness.^35^

The opposite was likewise evident. Several papers identified deficiencies in perceived clinician communication worsened emotional challenges and the moral conflict interpreters experienced.^30,32,35–37,39,40^ 18.9% of interpreters in the Australian survey reported difficult conversations were worsened when not conducted in a culturally appropriate manner.^35^

There were several examples of how different social and culture backgrounds and expectations of clinicians, interpreters and patients came together to complicate each party’s experience of interpretation. These included use of medical jargon or euphemisms,^30,32,35^ providers translating in the third person, or speaking directly to the translator^40^ or family member.^30,40^ Failure to allot sufficient time^30,35^ added to interpreter difficulties, leading to rushing and challenges in patient comprehension.^35^ Sometimes when physician questioning was deemed intrusive by patients/families, the interpreter would bear the brunt of this.^38^ Lack of provider empathy, sensitivity or engagement led to negative emotional effects.^30,35,37^ Spanish interpreters (*USA*) linked this with being comfortable in their perceived role, preferring to remain neutral if providers were empathic.^30^ Another study (*Multiple languages, Australia*) highlighted interpreters felt personal responsibility for communicating bad news sensitively, made challenging when perceived provider language was inadequate.^37^ Healthcare professionals in Australia recognised their communication styles could potentially cause interpreter discomfort.^36^

*Whatever, the provider said, we have to tell the patient. Professionally we really have to stick to the authenticity of what the provider said. But sometimes the provider’s word is a little bit too harsh . . . We have a lot of words in Chinese that are a little bit more polite than just telling them that you are going to die (Chinese-speaking Interpreter, USA)* ^32^

*It really do[es] help with the doctor’s attitude. . . if they are kind and compassionate. (Interpreter, Australia)* ^37^

*Maybe you feel uncomfortable with how someone is giving someone information or how they’re presenting it or how quickly they do it and you’ve got no voice to go, Can you stop? (Speech pathologist regarding interpreters, Australia)* ^36^

*So you’re just in the – the patient is in the background and you’re with the patient in the background so, sometimes you don’t have time to interpret everything. You ﬁnd yourself summarizing. And then, you are like, okay, so . . . ﬁrst of all, who am I to know what’s important in that conversation and what’s not, because I don’t have time to interpret everything. So you’re caught in that position where you have to say . . . she should probably know this. (Spanish interpreter, USA)* ^30^

Interpreters felt abandoned or misused by providers and were keen to be considered a valued healthcare team member, not ‘just an interpreter’.^34^ Discomfort with asking for clarification^38^ and being targets of provider frustration^38,39^ were reported.

### Working with patients and families

A prior relationship with the patient was cited as contributing to emotional distress. This was particularly relevant in small cultural communities such as Inuit interpreters,^38^ and reported in the Australian interpreter survey, in which 37.8% respondents identified this made discussions more difficult.^35^

*We know people from here. I felt heartbroken. I almost cried.* (*Inuit interpreter, Canada*)^38^

Patient and relative anger/distress or rudeness directed towards the interpreter also made some interpreters feel upset and disrespected^40^; a subset of survey respondents cited such behaviours made consultations more difficult.^35^ The need for Inuit interpreters to regulate conflict amongst patients, families and providers was deemed emotionally stressful.^38^

#### Interpreter experience and age

Three studies identified lack of interpreter experience, training, resources or preparation.^30,35,38^ In Australia, higher Secondary Traumatic Stress scores were identified in interpreters working less than 5 years and lower scores in those whose training specifically referenced interpreting difficult conversations.^35^ Experience was not associated with stress in the American survey.^33^Lack of training was described as a major challenge for Inuit interpreters, leading to feelings of being ‘forgotten’ and ‘unwanted’^38^; Spanish interpreters (*USA*) highlighted experience and palliative care-specific training were valuable.^30^

The Australian survey identified a significant age effect. Those aged 18–30 reported higher burnout and Secondary Traumatic Stress scores relative to older age categories.^35^ Females reported higher Compassion Satisfaction scores (69% relative to 59% males), but this was not statistically significant.^35^

## Recommendations to mitigate negative emotional effects

For part two of our research question, we identified recommendations included within papers, either as conclusions or direct suggestions from the studied population (Table 5).

**Table 5.** Summary of recommendations to improve interpreter experience.

Recommendation	Description	Terms used
Pre-briefing^30–36,38,40^	*Clinician-interpreter interaction prior to patient encounter to discuss the patient case to prepare emotionally and linguistically and clarify concepts/cultural nuances.*	Pre-encounter discussion^30^ Pre-encounter huddles^31^ Premeeting^32^ Meeting with interpreter before encounter^33–35,38^ Pre-briefing^36^ Briefing^40^
Debriefing^30,32–37,40^	*The act of reviewing and reflecting on a patient encounter, to process what went well/not well ± the emotions associated with this.*	Post-encounter discussion^30^ Debriefing^32,34–37,40^ Meet with interpreter after discussions^33^
Formal emotional support^35,36,40^	*Access to emotional support following distressing conversations. Includes counselling.*	Counselling^35^ Formal support^36^ Emotional support^40^
Interpreter training^30–33,35,37,38^	*Interpreter training to improve knowledge of palliative care concepts, ethical decisions, communication.*	
Provider training^32,33,35,36,38^	*Training for healthcare professionals in how to conduct palliative care conversations through an interpreter.*	

### Pre-briefing

Most studies identified the value of pre-briefing^30–36,38,40^ although some commented this was rarely done in practice.^36,40^ In surveys, 95% of American interpreters^33^ and 74.5% of Australian interpreters^35^ agreed healthcare professionals should meet with interpreters before discussions.

Pre-briefing offers opportunity for interpreter and provider to discuss clinical information,^30,32^ anticipated linguistic^30,40^ and cultural issues,^35^ and prepare interpreters emotionally.^30,31,38,40^ Some interpreters described this as allowing them to retain professionalism and neutrality and avoid ‘projecting emotional surprise’ (*Spanish; USA*).^30^ It further prompted more accurate translations^32,40^ and may be used to clarify interpreter role expectations.^33,34^

Pre-briefing was sometimes suggested as an interactive process between provider and interpreter, whereby interpreters could volunteer relevant cultural information.^34,35^ This may additionally improve provider-interpreter relationships.^31^ A QIP intervention including ‘huddles’ was described as an ‘opportunity to realise we are all human. . . this has reduced my past feelings of alienation (from physicians)’.^31^

*Going in cold is the biggest detriment. A short briefing prior to meeting the patient and family, in cases such as end-of-life, is central in allowing the interpreter to be at his/her best. (Interpreter, USA)* ^33^

### Debriefing

Debriefing was extensively recommended,^30,32–37,40^ mostly taking the form of a post-consultation meeting.^30,32–36^ Family debriefing was also mentioned.^37^ Like pre-briefing, debriefing occurred infrequently in clinical practice.^30,32,35–37,40^ 80% of American survey interpreter respondents recommended meeting with interpreters after discussions.^33^ 43.6% of Australian survey respondents felt debriefing would improve difficult conversations; 75% reported they would find debriefing or counselling helpful.^35^

Debriefing offered occasion to discuss what went well or not well,^35^ receive and provide feedback from and to healthcare professionals,^30,34^ and provide emotional support.^36,37^ Although not all studies elaborated on how to effectively debrief, Spanish interpreters (*USA*) identified value in being able to offer comment to providers on cultural or comprehension issues arising.^30^ Barriers included time,^32,36^ privacy^36^ and concerns regarding confidentiality within usual support networks.^40^

### Formal emotional support

Formal emotional support was referenced^35,36,40^ as another potential strategy to aid interpreters, although access to support was lacking.^35,38,40^ Counselling was considered useful in the Australian survey, particularly to express emotion (35.7%), gain closure (42.9%) and develop strategies to manage these conversations (57.1%).^35^ Of note, the interpreter’s commitment to confidentiality may compromise ability to access support to mitigate negative emotional effects.^36,38,40^

### Interpreter training

Interpreter training and experience was cited as a useful asset.^30–33,35,37,38^ 89%^33^ and 44.7%^35^ of respondents in American and Australian surveys, respectively, agreed interpreters needed more training. Those personally interested in more training did not differ by training, language or experience (*America*).^33^ As with formal emotional support, interpreters reported insufficient training in practice.^32,37,38^

A dialogue-based intervention showed significant improvements in confidence post-intervention and interpreter comments indicated benefits of dialogues and role-plays in preparing for difficult discussions, reporting greater purpose, confidence, collaboration and validation.^31^ Other suggestions for training were end-of-life communication,^32^ how to interpret these discussion types,^33,35^ understanding hospice logistics^32^ and improving linguistic knowledge of clinical information and ethical decisions.^38^ Inuit interpreters highlighted benefits of visual learning forms.^38^ Interpreters interviewed regarding the transition from oncology to palliative care inferred benefit by identifying emotional burdens associated with a lack of oncology-related training (*Multiple languages, Australia*).^36^ Spanish interpreters (*USA*) highlighted values of previous training, focusing on neutrality and personal ‘triggers’.^30^

*. . .dialogues were very helpful especially for the roleplaying. . . because we are doing actual role plays and this prepares us for situations with patients and providers. (Interpreter in dialogue-based intervention, USA)* ^31^

### Provider training

Provider training in how best to utilise and interact with interpreters was suggested by both interpreters^32,33,35,38^ and healthcare professionals.^36,38^ 81% of survey respondents felt physicians needed more training conducting end-of-life discussions through an interpreter (*USA*)^33^; 53.2% suggested more training for medical professionals in interacting with interpreters (*Australia*).^35^ Feedback from nurses, physicians and interpreters in Northern Quebec suggested improving provider-interpreter relationships with greater understanding of interpreter challenges (including moral dilemmas), inviting interpreter expertise and explaining provider rationale for challenging messages.^38^ Learning about cultural, spiritual and world views of Inuit communities was also encouraged.^38^ Focus on communication techniques was advocated, including clarity^38^; avoidance of vague language, medical jargon^33^ and humour.^33^

## Discussion

### Key findings and implications

Our rapid review is the first to synthesise data analysing the emotional effects on professional interpreters of engaging specifically in palliative care conversations. Our findings suggest that for many professional interpreters, facilitating conversations in palliative care could be difficult, stressful, distressing, and bring feelings of being overwhelmed, sad, lonely and guilty. These negative emotions correlate with findings from a systematic review exploring transferential dynamics and vicarious trauma in interpreters (trained and untrained) in a variety of emotive settings.^15^ As in our review, positive experiences were also reported.^15,16^

As in other settings,^15^ we found many emotions described related to moral and cultural conflict experienced by the interpreter, especially regarding their role and responsibility. Commitment to accuracy or acting as a neutral conduit was perceived as uncomfortable when provider language was untranslatable or culturally inappropriate. Accordingly, professional interpreters experienced the strain of responsibility to codes of ethics requiring accuracy, against an emotional desire to deliver culturally sensitive information as mediator or advocate. This was particularly challenging when their role was unclear.

Cultural norms may make discussion of death and ‘truth telling’ unacceptable in certain communities, with potentially harmful results.^41,42^ Kaufert and Putsch observes this conflict in Aboriginal Canadian interpreters, where cultural beliefs clash with other frameworks in provider-patient interactions, including autonomy in consent, and truth-telling in end-of-life decisions.^39,43^ Other literature demonstrates this conflict and role insecurity in emotionally-laden settings such as palliative care.^44,45^ We expand on these findings by isolating the emotions born from such challenges.

Clarifying the interpreter role could alleviate related distress. Healthcare professionals in this review volunteered myriad opinions regarding whether interpreters should maintain neutrality or be able to actively modify content.^36,38,39^ In a study of providers’ views on interpreters’ emotional support for patients, the interpreter’s role was viewed inconsistently, despite identifying that being both professional and human was necessary for them to demonstrate emotional support.^46^ As advocated by others, guidelines and institutions formalising realistic expectations of the interpreter’s role may go some way to alleviating role ambiguity.^43,44^ We advocate an expectation of professional interpreters being active participants, working together with providers to mediate important messages safely and sensitively. Further research is crucial to establish impact on patient safety and wellbeing.

The clinician’s responsibility in delivering culturally sensitive information in the patient encounter appears paramount to reducing interpreter moral conflict and subsequent distress, as supported by other literature.^44^ The collaborative relationship between interpreter and clinician is also important; working together to educate and prepare each other respectfully may reduce feelings of abandonment described in this review. A qualitative study of interpreters perceived collaboration and role-understanding as also imperative for patient safety.^47^ Other literature recommends providers and interpreters learn from each other and ‘co-evolve’.^48^ Hsieh and Hong^46^ identifies that provider identities are often mediated by interpreters; this means their emotional support towards patients is intertwined. Healthcare professionals demonstrate insight into challenges in cultural communication,^49^ which may improve ease of addressing this responsibility.

Interpreter and provider training to improve knowledge, cultural understanding, communication skills and collaborative working has potential to improve team relationships and patient care. That this was advocated by both interpreters and healthcare professionals indicates desire to drive change from both parties. The American survey reported high interest in training regardless of experience,^33^ and could therefore be relevant to all. Existing literature demonstrates the usefulness of provider and interpreter training in working together.^50,51^ Collaborative working alongside interpreters could be included in provider student curriculums. The dialogue-based intervention (*USA*) was well-received, particularly interactive roleplays,^31^ and may provide a framework for training. An education workshop for healthcare interpreters working in palliative care settings, including focus on personal challenges, improved interpreter understanding when piloted and could be a useful resource.^52^

Strategies such as pre-briefing, debriefing and training largely focus on provider-interpreter interaction, and are advocated in other research on interpreters.^15^ Pre-briefing was advocated in nine studies and across all represented countries, providing opportunity to discuss pre-encounter, allowing for role clarification and linguistic, cultural and emotional preparation. ‘Huddles’ or ‘brief, stand-up meetings’ within healthcare improved team communication, collaboration and staff satisfaction in a recent scoping review.^53^ Further attention into how best to conduct these would be beneficial, for example, a CHECK-IN tool has been hypothesised as a framework to guide collaborative huddles.^54^ Providing formalised support for professional interpreters following distressing consultations is necessary, with reassurance to reduce distress related to breaking confidentiality. This could be post-encounter debriefing or institutionally-provided counselling.

## Future research

Given the integral role of cultural factors affecting professional interpreter wellbeing, further research into influence of language (interpreted to and from) and cultural dynamics in different settings is warranted to elicit factors relevant to different communities. A broader assessment of impact is also integral – including social, cultural and religious consequences of interpreting such conversations.

Further research into optimising strategies to mitigate negative effects would be beneficial to address issues raised.

## Strengths and limitations

The rapid nature of our review is associated with methodological limitations.^55^ Limiting our search terms may have excluded relevant articles and increased risk of publication and country/language biases. Time and resource limitations necessitated most of the study selection process and thematic synthesis by one author (JH), although thematic reports were discussed with the whole research team. Our inclusion criteria sought to express common palliative care discussion topics, although defining all that constitutes a palliative care conversation is highly complex and beyond the scope of this review.

Although few studies focused on a specific language/cultural group,^30,32,38,39^ the remainder included either multiple languages or did not specify. It is therefore difficult to draw conclusions about influencing factors of language and culture as subgroup analyses were not conducted. We also need to be reflexively aware and situate our position as analysts when comparing and synthesising other researchers’ interpretation of participants’ reported emotions. We cannot assume interpretive authority over a study participant’s emotional experiences or the researcher’s descriptions and nor can we presume that will directly translate from one culturally specific context to another, even where similar terminology is used for example, distress, anger, sadness.^56^

Article comparability was limited by heterogeneity in methodology and aims/objectives, although most utilised qualitative data from focus groups or interviews. Findings were geographically limited, representing four developed countries (USA, Australia, Canada, UK), although two studies were based in minority cultural communities.^38,39^ These countries tend to support autonomous decision-making, which limits generalisability. However, a wide variety of languages and cultures were represented, with many consistent themes, suggesting there may be transferability to other contexts. Working in smaller Inuit and Aboriginal Canadian communities presented specific challenges, but with overlap of issues experienced in other communities.

There was a preponderance of female interpreters in several studies,^30,32–35,37^ although gender was not correlated with attitudes in American survey respondents^33^ and there were no statistically significant correlations in the Australian survey.^35^ Existing literature suggests burnout may be higher in female physicians,^57^ although studies in female interpreters found variable impact with burnout or Secondary Traumatic Stress.^58,59^ The gendered character of translation was also under-explored in the papers reviewed, which may explain the surprising lack of engagement with the emotional labour literature,^60^ besides a brief reference regarding the emotional labour entailed with talking about terminal diagnoses, palliative care, death and dying in one paper.^37^ Future research could explore the intersections of gender, culture and emotions in translation work.

## Conclusion

Professional interpreters are subject to an array of emotions when interpreting palliative care conversations, often alongside limited training and support. These findings indicate a need for interpreter role clarity, improved collaborative working in the healthcare team and formal guidelines to enhance provider-interpreter-patient experience. Employers should familiarise themselves with interpreter challenges in palliative care and strongly consider instituting recommendations identified in this review. Further research into how best to support our interpreter colleagues and the influence of cultural dynamics is essential. We hope this review will stimulate further interest in the interpreter’s role within palliative care and enable a platform for further research to give interpreters a voice.

## Supplemental Material

sj-pdf-1-pmj-10.1177_02692163231169318 – Supplemental material for The emotional effects on professional interpreters of interpreting palliative care conversations for adult patients: A rapid review Click here for additional data file. Supplemental material, sj-pdf-1-pmj-10.1177_02692163231169318 for The emotional effects on professional interpreters of interpreting palliative care conversations for adult patients: A rapid review by Jennifer A Hancox, Clare F McKiernan, Alice L Martin, Jon Tomas and John I MacArtney in Palliative Medicine

sj-pdf-2-pmj-10.1177_02692163231169318 – Supplemental material for The emotional effects on professional interpreters of interpreting palliative care conversations for adult patients: A rapid review Click here for additional data file. Supplemental material, sj-pdf-2-pmj-10.1177_02692163231169318 for The emotional effects on professional interpreters of interpreting palliative care conversations for adult patients: A rapid review by Jennifer A Hancox, Clare F McKiernan, Alice L Martin, Jon Tomas and John I MacArtney in Palliative Medicine
