# Supplementary material for: The emotional effects on professional interpreters of interpreting palliative care conversations for adult patients: A rapid review
Source: Palliat Med. 2023 Apr 24;37(7):931–46. doi: 10.1177/02692163231169318 (PMC10320707; doi:10.1177/02692163231169318)
Supplement: sj-pdf-1-pmj-10.1177_02692163231169318 – Supplemental material for The emotional effects on professional interpreters of interpreting palliative care conversations for adult patients: A rapid review [file sj-pdf-1-pmj-10.1177_02692163231169318.pdf]

Evidence Search Service
Results of your search request

What is the impact on interpreters of interpreting palliative care conversations?

ID of request: 33309
Date of request: 22nd December, 2021
Date of completion: 23rd December, 2021

Sources searched
CINAHL (30)
EMBASE (175)
MEDLINE (122)
PsycInfo (65)
PubMed (101)

Date range used (5 years, 10 years): All years.
Limits used (gender, article/study type, etc.): None
Search terms and notes (full search strategy for database searches below):

Databases searched: MEDLINE, EMBASE, CINAHL, PsycINFO, PubMED

For more information about the resources please go to: https://uhblibrary.co.uk/ .

Search History

#	Database	Search term	Results
1	Medline	(Palliative).ti,ab	68776
2	Medline	*"PALLIATIVE CARE"/ OR *"PALLIATIVE MEDICINE"/	34249
3	Medline	("end of life").ti,ab	25320
4	Medline	(Terminal).ti,ab	423836
5	Medline	*"TERMINAL CARE"/	22634
6	Medline	(Dying OR death).ti,ab	789554
7	Medline	*DEATH/	12411
8	Medline	("supportive care").ti,ab	17405
9	Medline	(1 OR 2 OR 3 OR 4 OR 5 OR 6 OR 7 OR 8)	1289264
10	Medline	(Interpreter*).ti,ab	3809
11	Medline	(Translator*).ti,ab	1900
12	Medline	(10 OR 11)	5626
13	Medline	(9 AND 12)	203
14	EMBASE	(Interpreter*).ti,ab	5267
15	EMBASE	(Translator*).ti,ab	2602
16	EMBASE	(14 OR 15)	7744
17	EMBASE	(Palliative).ti,ab	110353
18	EMBASE	("end of life").ti,ab	37421
19	EMBASE	(Terminal).ti,ab	476921
20	EMBASE	(Dying OR death).ti,ab	1129937
21	EMBASE	("supportive care").ti,ab	32895
22	EMBASE	*"PALLIATIVE TREATMENT"/	37829
23	EMBASE	*"TERMINAL CARE"/	20036
24	EMBASE	*DEATH/ OR *DYING/	24688
25	EMBASE	(17 OR 18 OR 19 OR 20 OR 21 OR 22 OR 23 OR 24)	1723043

26	EMBASE	(16 AND 25)	364
27	CINAHL	(Interpreter*).ti,ab	2387
28	CINAHL	(Translator*).ti,ab	736
29	CINAHL	(27 OR 28)	3055
30	CINAHL	(Palliative).ti,ab	38890
31	CINAHL	("end of life").ti,ab	22237
32	CINAHL	(Terminal).ti,ab	20610
33	CINAHL	(Dying OR death).ti,ab	208968
34	CINAHL	("supportive care").ti,ab	6723
35	CINAHL	*"PALLIATIVE CARE"/	27204
36	CINAHL	*"TERMINAL CARE"/	13939
37	CINAHL	*DEATH/	9659
38	CINAHL	(30 OR 31 OR 32 OR 33 OR 34 OR 35 OR 36 OR 37)	281703
39	CINAHL	(29 AND 38)	118
40	PsycINFO	(Interpreter*).ti,ab	3409
41	PsycINFO	(Translator*).ti,ab	1347
42	PsycINFO	(40 OR 41)	4623
43	PsycINFO	(Palliative).ti,ab	12124
44	PsycINFO	("end of life").ti,ab	9756
45	PsycINFO	(Terminal).ti,ab	15217
46	PsycINFO	(Dying OR death).ti,ab	90640
47	PsycINFO	("supportive care").ti,ab	1491
48	PsycINFO	*"PALLIATIVE CARE"/	13820
49	PsycINFO	*"TERMINALLY ILL PATIENTS"/ OR *"DEATH AND DYING"/	27982
50	PsycINFO	(43 OR 44 OR 45 OR 46 OR 47 OR 48 OR 49)	125261
51	PsycINFO	(42 AND 50)	105

Disclaimer
We hope that you find the evidence search service useful. Whilst care has been taken in the selection of the materials included in this evidence search, the Library and Knowledge Service is not responsible for the content or the accuracy of the enclosed research information. Accordingly, whilst every endeavour has been undertaken to execute a comprehensive search of the literature, the Library and Knowledge Service is not and will not be held responsible or liable for any omissions to pertinent research information not included as part of the results of the enclosed evidence search. Users are welcome to discuss the evidence search findings with the librarian responsible for executing the search. We welcome suggestions on additional search strategies / use of other information resources for further exploration. You must not use the results of this search for commercial purposes. Any usage or reproduction of the search output should acknowledge the Library and Knowledge Service that produced it.
