# Supplementary material for: The emotional effects on professional interpreters of interpreting palliative care conversations for adult patients: A rapid review
Source: Palliat Med. 2023 Apr 24;37(7):931–46. doi: 10.1177/02692163231169318 (PMC10320707; doi:10.1177/02692163231169318)
Supplement: sj-pdf-2-pmj-10.1177_02692163231169318 – Supplemental material for The emotional effects on professional interpreters of interpreting palliative care conversations for adult patients: A rapid review [file sj-pdf-2-pmj-10.1177_02692163231169318.pdf]

CASP checklist criteria for Qualitative Studies (Qu 1-9)

	**Rhodes**	**Silva**	**Norris**	**Martin**	**Kirby**	**Hordyk**	**Kaufert**	**Prentice**
1. Was there a clear statement of the aims of the research?	Y	Y	Y	Y	Y	Y	Y	Y
2. Is a qualitative methodology appropriate?	Y	Y	Y	Y	Y	Y	Y	Y
3. Was the research design appropriate to address the aims of the research?	Y	Y	Y	Y	Y	Y	Y	Y
4. Was the recruitment strategy appropriate to the aims of the research?	Y	Y	Y	Y	Y	Y	Y	Y
5. Was the data collected in a way that addressed the research issue?	Y	Y	Y	Y	Y	Y	Y	Y
6. Has the relationship between researcher and participants been adequately considered?	Y	Y	Y	Y	Y	Y	Y	CT
7. Have ethical issues been taken into consideration?	Y	Y	Y	Y	Y	Y	CT	Y
8. Was the data analysis sufficiently rigorous?	Y	Y	Y	Y	Y	Y	CT	Y
9. Is there a clear statement of findings?	Y	Y	Y	Y	Y	Y	Y	Y

Y = YES, N = NO, CT = CAN’T TELL

CASP checklist criteria for Qualitative Studies (Qu 10 with author comments)

	**10. How valuable is the research?**
Rhodes	Valuable. Discusses contribution of study in context of existing literature. Identified recommendations and areas for future research. Also comments on limited generalisability of findings (Spanish speaking only, one Midwestern state, relatively high interpreter certification level).
Silva	Valuable research - discussed contribution of study in context of current knowledge/understanding. Focus of future research identified. Noted limited generalisability of findings given single site study and use of experienced interpreters although continued until thematic saturation reached.
Norris	Valuable. Discusses study in context of existing knowledge, identifies new areas for research. Recognises limitations including - sample of interested volunteers from local chapter of national society (cannot be considered representative of all medical interpreters), high proportion from Asia and working in urban medical centres - may limit generalisability.
James	Valuable research. One of the largest studies of healthcare interpreters in Australia focusing on effects of difficult conversations. Describes in context of existing literature and offers future research directions.
Kirby	Valuable research. Findings discussed in context of current literature and several implications for policy and practice identified. Comments on limited generalisability - captures experience of participants in 2 hospitals in 1 Australian city; participants self-selected. Some focus on further research.
Hordyk	Valuable. Results very helpful locally in Inuit communities and actually informing policy. Recommendations made and future research directions identified. The Nunavik Health Board took findings from the study and in process of developing training based on these. Context of study in existing research described.
Kaufert,	Somewhat valuable in isolation. Valuable in the context of other works written by the author.<br>Conclusion comments more on the findings rather than identifying new areas of research/transferability of findings although does comment on research in context of other literature.
Prentice	Somewhat valuable as platform for larger studies. Some consideration to existing knowledge/understanding (although limited comparison to other literature in discussion). Very small sample size (n=5), recognises limitations, identifies need for larger study and further research areas.

AXIS (Appraisal Tool for Cross-sectional Studies) - **James**

Question		Yes	No	Don't know/ Comment
Introduction				
1	Were the aims/objectives of the study clear?	Y		
Methods				
2	Was the study design appropriate for the stated aim(s)?	Y		
3	Was the sample size justified?	Y		Not explicit but implied – represents 6.7% Australian population and one of largest studies of its kind
4	Was the target/reference population clearly defined? (Is it clear who the research was about?)	Y		
5	Was the sample frame taken from an appropriate population base so that it closely represented the target/reference population under investigation?	Y		Appropriate sample frame. Limitations recognise it is unclear how well the respondents represent the interpreters. Self-selection bias (voluntary), low uptake and not necessarily generalisable.
6	Was the selection process likely to select subjects/participants that were representative of the target/reference population under investigation?	Y		Although survey sent via interpreter companies – concerns regarding confidentiality, voluntary participation. Likely to be an issue in any interpreter survey.
7	Were measures undertaken to address and categorise non-responders?	Y		Follow up survey request at 2 & 4 weeks.
8	Were the risk factor and outcome variables measured appropriate to the aims of the study?	Y		Adjusted data for characteristics. Face validity and readability assessed,
9	Were the risk factor and outcome variables measured correctly using instruments/measurements that had been trialled, piloted or published previously?	Y		ProQOL 5 used – widespread use and well published.
10	Is it clear what was used to determined statistical significance and/or precision estimates? (e.g. p-values, confidence intervals)	Y		Mean/SD to describe the data. P values + CI used (although not specified in methods). No software package detailed. Does report linear regression used to assess impact on selected variables with crude (unadjusted) and adjusted estimates obtained.
11	Were the methods (including statistical methods) sufficiently described to enable them to be repeated?	Y		
Results				
12	Were the basic data adequately described?	Y		
13	Does the response rate raise concerns about non-response bias?	Y		Only 6.7% SA health interpreters responded – possibility of not being generalisable in discussion
14	If appropriate, was information about non-responders described?		N	
15	Were the results internally consistent?	Y		Generally consistent, although 1-2 interpreters not accounted for in training statistics
16	Were the results presented for all the analyses described in the methods?			
Discussion				
17	Were the authors' discussions and conclusions justified by the results?	Y		
18	Were the limitations of the study discussed?	Y		

Other				
19	Were there any funding sources or conflicts of interest that may affect the authors' interpretation of the results?		N	
20	Was ethical approval or consent of participants attained?	Y		

AXIS (Appraisal Tool for Cross-sectional Studies) – **Schenker**

**Question**		**Yes**	**No**	**Don't know/ Comment**
Introduction				
1	Were the aims/objectives of the study clear?	Y		
Methods				
2	Was the study design appropriate for the stated aim(s)?	Y		
3	Was the sample size justified?			DK - Not explicit but explained recruitment leading to sample size (8-week period for survey responses)
4	Was the target/reference population clearly defined? (Is it clear who the research was about?)	Y		
5	Was the sample frame taken from an appropriate population base so that it closely represented the target/reference population under investigation?	Y		
6	Was the selection process likely to select subjects/participants that were representative of the target/reference population under investigation?	Y		Convenience sample used – including current healthcare interpreters in a few interpreting groups (Texas, Nebraska, California) as well as advertising on social media/newsletter (latter may target certain populations)
7	Were measures undertaken to address and categorise non-responders?		N	Open for 8 weeks but no comment about how the demographics of the sample represent others
8	Were the risk factor and outcome variables measured appropriate to the aims of the study?	Y		
9	Were the risk factor and outcome variables measured correctly using instruments/measurements that had been trialled, piloted or published previously?	Y		Used previous qualitative study with interpreters as a basis for the survey development and added questions based on MDT project team. Piloted.
10	Is it clear what was used to determined statistical significance and/or precision estimates? (e.g. p-values, confidence intervals)	Y		
11	Were the methods (including statistical methods) sufficiently described to enable them to be repeated?	Y		Very extensively described.
Results				
12	Were the basic data adequately described?	Y		
13	Does the response rate raise concerns about non-response bias?			DK – non response rate not commented on by authors
14	If appropriate, was information about non-responders described?		N	
15	Were the results internally consistent?	Y		Mostly, yes. Data not shown for interpreter characteristics and attitude interpretation, but comment made that no characteristics were associated with attitudes. Typo noted in Table 2.
16	Were the results presented for all the analyses described in the methods?	Y		
Discussion				
17	Were the authors' discussions and conclusions justified by the results?	Y		
18	Were the limitations of the study discussed?	Y		
Other				
19	Were there any funding sources or conflicts of interest that may affect the authors' interpretation of the results?		N	Received funding but should not affect results – one doctor received payment for development of educational materials. Other grants declared but appear appropriate.
20	Was ethical approval or consent of participants attained?	Y		

QI-MQCS (Quality Improvement Minimum Quality Criteria Set) - **Goldhirsch**

Domain	Achieved? Y/N/DK	Comment
1. Organisational motivation	Y	Justified on both a local and international level
2. Intervention rationale	Y	Explains purpose of intervention and links to intended effects
3. Intervention description	Y	Describes the intervention clearly and in detail
4. Organisational characteristics	DK	Whilst the general features of the hospital were not commented on, the hospitals were named, and anecdotal evidence of employee experience mentioned – in addition to a description of the interprofessional team working in the palliative care department
5. Implementation	Y	Utilised findings from baseline assessment to design intervention, with input from very experienced team with knowledge of interpreters
6. Study design	Y	Pre & post course questionnaires to assess confidence
7. Comparator	Y	Describes interpreter group pre-intervention and limited resources available
8. Data source	Y	Questionnaire an adapted confidence questionnaire developed for the Serious Illness Care Program for Ariadne Labs designed for internists (frequently used but non-validated). Pre & post course questionnaires given
9. Timing	Y	Timeframe of intervention and assessment described, although exact dates not included. Discussion reports ongoing roll out of intervention and dates for this.
10. Adherence/fidelity	Y	Percentage of attendees who completed assessments/the full course, and description of how data was managed.
11. Health outcomes	DK	Unclear, but there is a reference to clinicians noting importance of maximising interpreter performance in conclusion, especially during family meetings. Could infer improved patient outcomes but not explicitly stated.
12. Organisational readiness	Y	Lessons learned covers this
13. Penetration/reach	Y	Describes intervention offered in 2 hospital sites as part of professional medical interpreter continuing education series. Described in outcomes as proportion of interpreters who completed the questionnaires relative to number completing the course.
14. Sustainability	Y	Ongoing roll out of the programme described

15. Spread	Y	Summary curriculum included (Table 1) and full materials, curriculum, and reference materials available on request
16. Limitations	N	Not explicitly reported, although missing data stated and described how this was handled. Certainly, limitations present, but remains a useful resource and relevant to our study.

Y = YES, N = NO, DK = Don’t Know
